# Association of Food Addiction With Obesity in Young Adults Belonging to Urban and Rural Populations

**DOI:** 10.7759/cureus.74370

**Published:** 2024-11-24

**Authors:** Setu Sukesh, G Vishnuvardhan, M Vidhyavathi

**Affiliations:** 1 Psychiatry, Rajarajeswari Medical College & Hospital, Bengaluru, IND

**Keywords:** food addiction, obesity, yale scale, yfas, young adults

## Abstract

Background

Food addiction (FA), where an individual displays a loss of control over the consumption of calorie-dense foods (refined carbohydrates, fats), is proposed to be like substance-use disorders with the experience of cravings, reduced control over intake, increased impulsivity, and altered reward-sensitivity. FA may also be associated with obesity. This study aimed to determine the prevalence of FA in urban and rural areas, and the proportion of obesity in young adults with FA.

Methods

This cross-sectional study involved 480 participants (240 each from urban and rural populations). Sociodemographic information and anthropometric measurements were recorded. The Yale Food Addiction Scale (YFAS), designed to assess signs of addictive-like eating behaviour, was used to determine which individuals had FA.

Results

Young adults (208 males and 272 females) with an average age of 21.6 years were included. FA was found in 40 (16.7%) urban and 28 (11.7%) rural subjects. Among 68 subjects with FA, 48 (70.5%) were obese and 20 (29.4%) were non-obese. The most frequent FA symptom in the urban and rural subjects was a persistent desire to eat or a repeated failure to quit in 228 (95%) and 192 (80%) subjects, respectively, followed by the development of tolerance in 108 (45.0%) and 60 (25%) subjects, respectively.

Conclusion

Food addiction is common in young adults and is higher in urban than in rural populations. The association of FA with indicators of obesity is high. Studying the prevalence of FA in different population groups can also enable a better understanding of the concept and its pathophysiology.

## Introduction

Obesity is the abnormal or excessive accumulation of fat that presents a health risk. Hypertension, metabolic syndrome, dyslipidemia, type 2 diabetes mellitus (T2DM), and cardiovascular disease (CVD) are a few of the numerous obesity-related co-morbidities. Obesity is a polyfactorial disorder that usually results from a complex interaction between an individual's metabolism and environmental factors that lead to an energy surfeit [1]. Contributing to this positive energy balance are overeating (spurred on by the easy availability of energy-dense and palatable foods) and reduced physical activity (due to increased mechanization of manual tasks, increased desk jobs, and the work-from-home concept) [1]. While some people manage to overcome the urge to overeat and maintain a healthy weight, a few individuals show a preference for energy-dense foods [2,3].

The concept of “food addiction” (FA) has been in the media for a few years and it is gaining the interest of the scientific community of late. Food addiction is a syndrome denoted by an irresistible desire for the consumption of palatable foods that may be highly refined, energy-dense, containing high salt, sugar, and fat, mainly for reward-related consumption, regardless of its energy-producing potential [3]. Similarities between FA and substance use disorders (SUD), like, operant conditioning, effects of mood and stress, cue reactivity, craving, and impulsivity, support food as an addictive substance [3]. To aid in the diagnosis of ‘food addiction’ the Yale Food Addiction Scale (YFAS) has been developed and validated [4].

Food addiction, though not recognized in the Diagnostic and Statistical Manual (DSM) of the American Psychological Association yet, has been shown to activate the same neural reward circuits (dopaminergic and opioid systems) as drugs of abuse in animal models [5].

Thus, it can be hypothesized that obesity is driven by the subject’s dependence on food like a person's dependence on substances of abuse, stimulating a pathway contributing to sustained overeating and obesity. There may be a likely relationship between behavior and weight gain [5]. It, therefore, may be necessary to study the effect of psychological, behavioral, cognitive, and physiological factors in the food addiction construct, thus implying the possibility of preventing and treating obesity [5-7].

A recent study, using the Indian cut-off of BMI of ≥25kg/m^2^, found the prevalence of obesity in India to be 40.3% [1]. The prevalence of obesity was significantly higher among urban residents compared to rural residents [1]. The burden of obesity and non-communicable diseases continues to grow in India, where the prevalence of an overweight and obese population is increasing faster than the world average. 

A recent study recruited 415 Indians to complete an online survey using self-report measures assessing FA (YFAS), eating disorder psychopathology (Eating Disorder Examination Questionnaire (EDE-Q)), health-related quality of life (12-item Short Form survey (SF-12)), and depression (Patient Health Questionnaire-2 (PHQ-2)). The FA symptom mean was 3.53 (SD=1.90); 32.5% (n=129) met the FA clinical threshold on the YFAS. Groups categorized with and without FA on the YFAS did not differ significantly in sex or BMI. YFAS scores were significantly correlated with greater frequency of binge eating, higher severity scores on all EDE-Q subscales, higher depression, and poorer functioning scores [8].

Another online YFAS questionnaire-based study [9] conducted using convenience sampling in India having 376 respondents (median age 19 years, 42.8% males), found the rate of occurrence of food addiction was 13.3%. Persistent desire or repeated unsuccessful attempts to quit was the most common symptom domain endorsed. The weight and BMI were higher in the food addiction group as compared to the non-food addiction group [9]. 

There are few studies [8,9] done on food addiction in India, and they were either online surveys or conducted in study samples not representative of Indian populations in terms of differing levels of physical activity, body fat composition, the prevalence of obesity and socio-economic groups, and real-time anthropometric measurements. This is of emerging importance as the prevalence of obesity and associated disorders has shown a rapid increase in rural areas and a larger relative increase in obesity prevalence is predicted in rural areas, in comparison to urban areas [10].

This research was undertaken with an objective to determine the prevalence of food addiction, to assess the relationship of food addiction with indicators of obesity, and to determine the presence of symptoms of food addiction amongst young adults residing in urban and rural areas.

## Materials and methods

This cross-sectional study was carried out in Bengaluru, India, after obtaining ethical clearance from the Institutional Ethical Committee of Rajarajeswari Medical College and Hospital, Bengaluru. The protocol was an Indian Council of Medical Research (ICMR) approved STS 2022 (Short-Term-Studentship) project. Data collection was done for a period of three months, between August and October 2022. Based on the literature review, in a previous study conducted by Hauck et al., it was found that the proportion of subjects who had food addiction was 7.9% [6]. In the present study, expecting similar results considering 5% absolute precision and 95% confidence level, the sample size worked out to be a minimum of 120 patients. Four hundred and eighty subjects, of both genders, aged between 18 and 25 years, 240 residing in urban areas and 240 residing in rural areas, were included in the study. Written informed consent was obtained from all the subjects included in the study. Patients diagnosed with other eating disorders like anorexia nervosa, bulimia nervosa, etc.; pregnant women and breast-feeding mothers; patients on medication that can alter weight or food intake, such as steroids, neuroleptics, selective serotonin reuptake inhibitors (SSRIs), etc.; patients with a history of a major illness/hospitalization; and patients with a history of psychiatric disorders in the last 6 months were excluded from the study. In a pre-structured questionnaire, the socio-demographic details, weight, height, and waist circumference were measured and recorded using standardized instruments and protocols [11]. A BMI of 18.5 kg/m2 to < 25 kg/m2 was considered non-obese and a BMI ≥25 kg/m2 was considered as obese as per cut-offs prescribed for an Indian population. The waist-to-height ratio, which indicates the risk of developing metabolic and cardiovascular complications, was also calculated. A waist-to-height ratio of < 0.52 was considered normal. Abdominal obesity was considered when the waist circumference was ≥90 cm in males and ≥80 cm in females [1,11].

The YFAS was explained to the subjects through a translation into the local language (Kannada) done by two bilingual doctoral-level experts who had previously not seen the instrument. This was done to ensure and facilitate a better understanding of the questionnaire by the urban and rural population sample. Scoring of the YFAS was done according to the procedure followed by Gearhardt et. al., which evaluates the presence of substance dependence criterion [4, 12]. The Yale Food Addiction Scale is a 25-item self-report measure that has been developed to identify those who are most likely to exhibit markers of substance dependence with the consumption of high-fat/high-sugar foods and salty snacks in the past 12 months. The scale questions fall under specific criteria that resemble the symptoms of substance dependence as stated in the Diagnostic and Statistical Manual of Mental Disorders (DSM) IV-R and operationalized in the Structured Clinical Interview for DSM-IV Axis I Disorders. The questionnaire has 16 Likert-rated items, nine yes/no-rated items, one multiple-choice item, and one open-ended question. Based upon the responses, the individual is classified as having either food addiction or not. The diagnosis of food addiction is based upon the presence of at least three of seven criteria, along with the presence of the clinical impairment criterion [4, 12].

Descriptive statistics were used to represent the nominal, ordinal, and scale data. Categorical data was represented in the form of Frequencies and proportions. The chi-square test or Fischer’s exact test (for 2x2 tables only) was used as a test of significance for qualitative data. Continuous data was represented as mean and standard deviation. The independent t-test was used as a test of significance to identify the mean difference between two quantitative variables. MS Excel and MS Word were used to obtain various types of graphs (Microsoft Corporation, Redmond, USA). A p-value (probability that the result is true) of <0.05 was considered statistically significant after assuming all the rules of statistical tests. Statistical software MS Excel and SPSS version 22 (IBM Corp., Armonk, USA) were used to analyze data.

## Results

A study population of 480 subjects (240 residing in rural areas and 240 in urban areas) was chosen by random sampling. The demographic details of the study subjects are shown in Table 1. There were 208 (43.3%) males and 272 (56.6%) females among the 480 subjects. Among all the study subjects, 360 (75%) had a non-obese BMI while 84 (35%) out of 240 urban subjects and 36 (15%) of 240 rural subjects were obese, having a BMI ≥25kg/m^2^. A normal waist circumference was seen in 364 (75.8%) of the study population, while 76 (31.66%) of the urban and 40 (16.66%) of the rural subjects had an increased waist circumference that indicated abdominal obesity. A waist-to-height ratio of >0.52 was found among 140 (29.1%) of all study subjects. Food Addiction (FA) was seen in 68 (14%) out of the total 480 study subjects. 28 (13.5%) of the 208 males and 40 (14.7%) of the 272 female subjects had FA. Out of 240 urban subjects 40 (16.7%) subjects and 28 (11.7%) out of 240 rural subjects were found to have FA (Table 1).

**Table 1:** Demographic Details of Study Subjects

	Total n=480		Urban Subjects n=240		Rural Subjects n=240	
	n	%	n	%	n	%
Age in years (Mean, SD)	21.6, 2.09		21.52, 2		21.8, 2.2	
Gender						
Males	208	43.3	96	40	112	46.6
Females	272	56.6	144	60	128	53.3
BMI Kg/m^2^						
Non Obese (18.5 to <25)	360	75	156	65	204	85
Obese (≥25)	120	25	84	35	36	15
Waist Circumference						
<90 cm in males and <80 cm in females	364	75.8	164	68.33	200	83.3
≥90 cm in males and ≥80 cm in females (Abdominal obesity)	116	24.1	76	31.66	40	16.6
Waist to Height Ratio						
<0.52	340	70.8	160	66.66	180	75
≥0.52 (increased risk for developing metabolic and cardiovascular complications)	140	29.1	80	33.33	60	25
Food Addiction						
Not Present	412	85.8	200	83.3	212	88.33
Present	68	14.1	40	16.6	28	11.66

Table 2 shows details about the subjects with and without food addiction. Among the 68 subjects with food addiction, 40 (58.8%) were from the urban population and 28 (41.1%) were from the rural population. Females constituted 40 (58.8%)out of 68 subjects of those with food addiction. About 48 (70.5%), 52(76.4%), and 56 (82.3%) of those with food addiction had a BMI ≥ 25 Kg/m^2^, a waist circumference ≥90 cm in males and ≥80 cm in females, and a waist-to-height ratio ≥0.52, respectively.

**Table 2:** Details of Subjects With and Without Food Addiction

	Subjects with Food Addiction n=68		Subjects without Food Addiction n=412	
	N	%	N	%
Urban Population (n=240)	40	58.8	200	48.5
Rural Population (n=240)	28	41.1	212	51.4
Gender				
Males (n=208)	28	41.1	180	43.6
Females (n=272)	40	58.8	232	56.3
BMI Kg/m^2^				
Non Obese (18.5 to <25)(n=360)	20	29.4	340	82.5
Obese (≥25)n=120	48	70.5	72	17.4
Waist Circumference				
<90 cm in males and <80 cm in females (n=364)	16	23.5	348	84.4
≥90 cm in males and ≥80 cm in females(Abdominal obesity) (n=116)	52	76.4	64	15.5
Waist to Height Ratio				
<0.52 (n=340)	12	17.6	328	79.6
≥0.52 (increased risk for developing metabolic and cardiovascular complications) (n=140)	56	82.3	84	20.3

The most frequent FA symptom in the urban and rural subjects was a persistent desire to eat or a repeated failure to quit in 228 (95%) and 192 (80%) subjects, respectively, followed by the development of tolerance in 108 (45.0%)and 60 (25%) subjects, respectively. Amongst the symptom domains of food addiction, it was found that there was a statistically significant difference with respect to Domain II (p-value 0.025) and Domain VI (p-value 0.021) between urban and rural populations. There was no statistically significant difference found between urban and rural populations with respect to other domains (Table 3).

**Table 3:** Comparison of Responses to the YFAS questionnaire items/various Domains of Food Addiction among the Urban and Rural subjects * A p-value of <0.05 was considered statistically significant. YFAS: Yale Food Addiction Scale

YFAS questionnaire items/Domains of food addiction	Urban n (%) n=240	Rural n (%) n=240	p-value*
Domain I (substance taken in larger amount) Answered as -No	208 (86.7%)	212 (88.3%)	
Domain I (substance taken in larger amount) Answered as -Yes	32 (13.3%)	28 (11.7%)	1.00
Domain II (Persistent desire or repeated unsuccessful attempts to quit) Answered as -No	12 (5%)	48 (20%)	
Domain II (Persistent desire or repeated unsuccessful attempts to quit) Answered as -Yes	228 (95%)	192 (80%)	0.025
Domain III (Much time/activity to obtain, use, recover) Answered as -No	184 (76.7%)	196 (81.7%)	
Domain III (Much time/activity to obtain, use, recover) Answered as -Yes	56 (23.3)	44 (18.3%)	0.654
Domain IV(Important social, occupational activities given up or reduced) Answered as- No	180 (75%)	196 (81.7%)	
Domain IV (Important social, occupational activities given up or reduced) Answered as -Yes	60 (25%)	44 (18.3%)	0.507
Domain V (Use continues despite knowledge of adverse consequences) Answered as -No	184 (76.7%)	188 (78.3%)	
Domain V (Use continues despite knowledge of adverse consequences) Answered as -Yes	56 (23.3)	52 (21.7%)	1.00
Domain VI (Tolerance) Answered as - No	132 (55%)	180 (75%)	
Domain VI (Tolerance) Answered as - Yes	108 (45%)	60 (25%)	0.021
Domain VII (Withdrawal symptoms) Answered as - No	204 (85%)	212 (88.3%)	
Domain VII (Withdrawal symptoms) Answered as - Yes	36 (15%)	28 (11.7%)	0.789
Domain VIII (causes clinically significant impairment or distress) Answered as - No	200 (83%)	212 (88.3%)	
Domain VIII (causes clinically significant impairment or distress) Answered as -Yes	40 (16.7%)	28 (11.7%)	0.620

## Discussion

Obesity is considered one of the most significant risk factors for developing numerous diseases. A study by Oliveira J et. al, suggests that FA may lead to compulsive overeating that could lead to obesity [13]. 

In the present study, the prevalence of FA among the urban and rural young adult populations was noted as 40 (16.7%) and 28 (11.7%), respectively. The authors in the initial study that outlined the YFAS's development showed appropriate consistency (Kuder- = Richardson's 0.92) [13]. The original YFAS recorded prevalence rates ranging from 10.0% to 15.8% [13]. 

This study included 480 subjects, 240 subjects from rural areas and 240subjects from urban areas. The mean age of the urban and rural participants was 21.52±2 and 21.80±2.2 years, respectively. In a study by Wattick RA et al. [14], where they studied the influences on FA among young adults, it was revealed that the mean age of the participants was found to be 22.03 ± 5.15 years, which lines up with the current study. In another study, conducted in young adults of Taiwan [15], to assess FA in young adults. It was reported that the average age was 26.9 years. Studies [16,17] have also found that the role of stress, trauma, and adversity particularly early in life can work as a contributing factor in both drug addiction and FA. Also, foods that are rich in fats and sugars stimulate our reward circuits, which makes the food more pleasurable and causes cravings, leading to addiction. 

Eighty-four subjects (35%) and 36 (15%) subjects were recorded to be obese in urban and rural areas, respectively. In the present study, it was seen that more people from urban areas were overweight when compared to rural subjects, though the difference is narrow. A study by Thapa R et al. [18] showed similar results when the urban-rural differences in overweight and obesity were studied. They reported overweight and obesity were higher in the urban areas (35.4%) in comparison with rural areas (27.9%). Although it is currently believed that 40-70% of obesity and overweight is attributed to genetics, however, on the other hand, according to Kirchengast S et. al, [19], it can be characterized by rapid urbanization, with the mechanization of jobs, better transportation options, easy accessibility to the food with high energy density, and decreased physical activity. 

In the current study, FA was observed in 40 (16.7%) and 28 (11.7%) subjects, in urban and rural populations, respectively. This can be attributed to the sedentary lifestyle and overeating due to high stress levels among the urban population. These subjects were advised to visit the Psychiatry Outpatient Clinic for further evaluation and advice. 

It was observed that 58.8% (40 out of the 68) subjects with food addiction were female. Akin to the above results, a study by Jahrami H et al. [20] showed that FA was slightly more prevalent among female participants (20.3%) in comparison with the male participants (17.4%), though it was not statistically significant. In one of the studies [21] among young adults, it was revealed that women reported uncontrolled eating more than men when in stress, depression, or anxiety. A study by Hussenoeder FS et al., [22] indicated that only women were influenced by anxiety and subsequent FA. Another study on young people's eating patterns by Abdalla et al. [23] concluded that females were more likely to exhibit abnormal eating habits.

Of the studied subjects, FA was observed in 20 (5.6%) and 48 (40%) individuals with normal BMI and overweight, respectively. Hence, individuals with overweight are more addicted to food in the present study. A study [24] analyzing FA in a group of young people showed that the prevalence of FA was high among overweight and obese patients (42.2%). A significant link between FA and obesity was demonstrated by another study [25], which found that 54.1% of overweight patients had FA. 

Despite the growing scientific interest in FA, there has been little progress in the development of evidence-based treatments. Only eight research projects on FA treatment were identified in a systematic review by Cassin et al. [26], who came to the conclusion that there is insufficient evidence to recommend any particular intervention. Lifestyle modification (diet and physical activity advice), pharmacotherapy (combination of naltrexone and bupropion which acts on hypothalamic and reward circuits, and pexacerfont which is a corticotropin-releasing factor 1 (CRF1) antagonist), cognitive behavior therapy, psychobiotics, low carbohydrate ketogenic therapy, self-help groups, virtual reality (VR), and neuromodulation techniques are suggested for incorporation into the management of FA [27]. In a group of 178 adults enrolled in a 14-week behavioral weight-loss program, Chao et al., [28] examined changes in weight and FA symptoms, demonstrating that losing weight could be beneficial in subjects with FA.

Waist circumference and weight: height ratio was measured among the subjects and it was found that the 16 subjects (9.8%) with normal waist circumference and 24 subjects (31.6%) subjects with abnormal waist circumference (indicating abdominal obesity) had FA in the urban participants. Among the rural participants, only the participants with abnormal waist circumference and waist: height ratio (implying increased risk for developing metabolic and cardiovascular complications) had FA (28 (70%) and 28 (46.7%), respectively (p-value <0.001)). Abdalla MMI et al. [23] examined the relationship between abnormal eating habits, BMI, and waist-to-height ratio among Malaysian students. They discovered that the risk of abnormal eating habits increased with both BMI and waist-to-height ratio. The study also revealed a substantial significant relationship between abnormal eating habits and the waist-to-height ratio. The subjects who were found to have an obese BMI, a waist circumference that was above the normal cut-off, and/or a waist-to-height ratio that indicated increased cardiovascular risk were advised to visit the Internal Medicine Outpatient Clinic of our hospital for further evaluation and advice.

The frequency of symptoms according to DSM IV in our study was found to be a persistent desire or repeated unsuccessful attempts to quit both in urban (228 (95.0%)) and rural populations (192 (80.0%)), followed by the development of tolerance (108 (45%) and 60 (25%), in urban and rural subjects, respectively). A Mexican study by Cura-Esquivel I et al. [29] revealed that the most commonly observed symptoms among the subjects were a persistent desire or repeated unsuccessful attempts to cut down consumption (46%) followed by withdrawal from or reduction of important social, occupational, or recreational activities, due to addiction (38.1%) and persistent use despite knowledge of adverse consequences (37.1%). In a study by Valtier MCG et al, tolerance was observed in the majority of the subjects (85%) [30]. The findings of the above study were quite consistent with the current study. 

Hitherto, studies done in India on FA were either online surveys or conducted in study samples not representative of Indian populations in terms of differing levels of physical activity, body fat composition, prevalence of obesity, and socio-economic groups. There was no real-time or manual check for anthropometric measures of obesity [8,9]. But most importantly, a study and comparison of the prevalence of food addiction in urban and rural populations has not been previously done. The present study was therefore, conducted, to assess the prevalence of food addiction in young adults residing in rural and urban areas, representing different socio-economic strata and levels of physical activity, along with a real-time assessment of anthropometric indices of obesity, working towards a continual improvisation from the past studies. Some limitations in the present study are as follows: the sampling was in urban and rural areas and was restricted to areas in and around the field practice area of the hospital. A more inclusive study, including populations from different geographical and climatic regions, would have given more generalizable findings. Furthermore, assessing the correlation between body fat percentage and food addiction would also add value to the data available on obesity and food addiction. We also did not assess the convergent validity, divergent validity, test-retest reliability, or concurrent validity of the Kannada version of the YFAS questionnaire. We were not able to look at the temporal stability of food addiction and did not get the chance to evaluate other related determinants like physical activity, body fat composition, and calorie or fat intake. Additionally, the relatively shorter study period was noted as a constraint in the evaluation of various determinants of food addiction and obesity. This is primarily due to the study's exploratory nature and the vast scope for further research in this field. We are of the belief that further rigorous research is required to draw firm conclusions.

## Conclusions

Although there may be a statistically insignificant difference, the prevalence of FA was found to be higher in the urban than in the rural subjects. Higher body weight, BMI, waist circumference, and waist-to-height ratio were found to be correlated with FA. Future research may examine the plausible connection between FA biomarkers and the aetiopathogenesis of obesity. Research on neurobiological mechanisms underlying the emergence of addiction to particular food products as well as techniques to manage cravings related to FA in order to improve treatment plans and avoid the onset of obesity would be beneficial. To the best of our knowledge, this is one of the first studies in India to study the relation of food addiction to anthropometric indicators of obesity in rural and urban settings. There are very few research studies in India that have examined FA in relation to various population categories. Therefore, in order to buttress and augment the existent knowledge about the neuro-psycho-biological entity of food addiction, large-sized, follow-up population studies need to be undertaken in the country.
